# Anti atherosclerosis effect and mechanism of a novel curcumin analogue CACN136: regulating macrophage M1/M2 polarization and lipid metabolism

**DOI:** 10.3389/fphar.2025.1632647

**Published:** 2025-06-25

**Authors:** Qianjiao Zhao, Yueting Zhong, Zheng Li, Jia Tang, Chao Pi, Wenwu Zheng, Peng Shi, Ying Zuo, Jun Jiang, Yan Yang, Shifeng Chu, Yumeng Wei, Ling Zhao

**Affiliations:** ^1^ Key Laboratory of Medical Electrophysiology, Ministry of Education, School of Pharmacy, Southwest Medical University, Luzhou, China; ^2^ Luzhou Key Laboratory of Traditional Chinese Medicine for Chronic Diseases Jointly Built by Sichuan and Chongqing, The Affiliated Traditional Chinese Medicine Hospital, Southwest Medical University, Luzhou, Sichuan, China; ^3^ Central Nervous System Drug Key Laboratory of Sichuan Province, School of Pharmacy, Southwest Medical University, Luzhou, Sichuan, China; ^4^ Department of Cardiology, The Affiliated Hospital, Southwest Medical University, Luzhou, Sichuan, China; ^5^ Department of Comprehensive Medicine, The Affiliated Traditional Chinese Medicine Hospital, Southwest Medical University, Luzhou, Sichuan, China; ^6^ Department of Thyroid Surgery, The Affiliated Hospital, Southwest Medical University, Luzhou, Sichuan, China; ^7^ Tate Key Laboratory of Bioactive Substances and Functions of Natural Medicines, Institute of Materia Medica and Neuroscience Center, Chinese Academy of Medical Sciences and Peking Union Medical College, Beijing, China

**Keywords:** atherosclerosis, curcumin analog, macrophage, M1/M2, lipid metabolism, CACN136

## Abstract

**Introduction:**

Curcumin has been found to inhibit atherosclerosis. However, its poor stability and low activity severely limit its further application. To overcome the shortcomings of curcumin, our team successfully designed a novel curcumin analog, CACN136. This study aims to explore the anti-atherosclerosis effects of CACN136 and its mechanisms.

**Method and Result:**

Oil Red O staining results showed that CACN136 significantly improved atherosclerosis plaques in the aorta and aortic root of ApoE-/- mice. RNA sequencing analysis (RNA-seq) indicated that CACN136 inhibits atherosclerosis by regulating lipid metabolism and inflammation-related pathways. *In vitro*, CACN136 significantly upregulates the mRNA and protein expression of iNOS and Arg1 in LPS-induced RAW264.7 cells. In ox-LDL-induced RAW264.7 foam cells, CACN136 significantly reduced free cholesterol and total cholesterol levels, and the levels of ABCA1, CD36, and SRA1 mRNA and protein were significantly altered. *In vivo*, CACN136 significantly reduced lipid and inflammatory levels, with superior safety and efficacy compared to the same dose of simvastatin.

**Discussion:**

CACN136 improves atherosclerotic plaque by regulating macrophage polarization and lipid metabolism, suggesting that CACN136 may be a promising new drug for the treatment of atherosclerosis.

## 1 Introduction

Cardiovascular disease (CVD) is a common and serious illness that poses a significant threat to human life and health, accounting for over 31% of global deaths (Roth et al., 2020). It ranks first in terms of incidence and mortality rates in countries such as China, Europe, the United States, and other developed nations (The, 2023; Lv et al., 2024; Perry et al., 2023). Currently, atherosclerosis has emerged as the major etiology underlying various CVDs (Nedkoff et al., 2023), including hypertension, coronary heart disease, and acute myocardial infarction. Atherosclerosis is responsible for approximately 17.6 million deaths annually, posing a severe threat to human health (Roth et al., 2020).

Atherosclerosis is a complex dynamic pathological process that primarily affects large and medium-sized arteries (Hou et al., 2023; Jing et al., 2023). In the early stages of atherosclerosis, endothelial cells attract monocytes to the arterial wall through chemokine-receptor interactions and increased expression of intercellular adhesion molecules ICAM-1 and vascular cell adhesion molecule VCAM-1 (Pickett et al., 2023; Cybulsky and Gimbrone, 1991). Once monocytes migrate to the vessel wall, they differentiate into macrophages, which further polarize into M1 and M2 phenotypes under chronic inflammatory stimulation. At the same time, the increased activity of cholesterol transport pathways leads to a large uptake of cholesterol by pro-inflammatory M1 macrophages. In addition, the expression of receptors (including scavenger receptors) on macrophages increases. With the promotion of scavenger receptor activity, cholesterol accumulated in foam cells will lead to inflammation and aggravate the progress of atherosclerosis (Al-Hawary et al., 2023). Ultimately, lipid deposition forms heterogeneous AS plaques, and inflammation renders the plaques vulnerable, with thin fibrous caps, enlarged necrotic cores, and increased susceptibility to rupture and expose pro-thrombotic material, leading to thrombosis and arterial occlusion. Thus, the progression of atherosclerosis is consistently associated with macrophage lipid transformation and chronic inflammation. Currently, lipid-lowering drugs (including statins and PCSK9 inhibitors) are the standard therapeutic agents for atherosclerosis. Although statin therapy has been shown to reduce the risk of atherosclerosis events (Ference et al., 2017), long-term use of statins may lead to liver enzyme abnormalities, muscle toxicity, and diabetes mellitus, with poor patient compliance (Björnsson, 2017), and PCSK9 inhibitors also have the inherent drawbacks of monoclonal antibody drugs, such as high dosage requirements and frequent administration (Maningat et al., 2013). Therefore, given the shortcomings of current first-line clinical drugs, it is particularly necessary and urgent to develop safe and effective new anti-atherosclerosis drugs.

In recent decades, researchers have endeavoured to search for potential anti-atherosclerotic agents among natural compounds. Among them, curcumin, a naturally occurring bioactive polyphenol, has been extensively studied in anti-atherosclerosis due to regulating cholesterol homeostasis and macrophage polarization (Menon and Sudheer, 2007; Momtazi-Borojeni et al., 2019; Lin et al., 2020). Although curcumin has shown certain advantages and a good safety, it was severely restricted for further development and application due to its poor stability and low activity. Researchers are simultaneously searching for analogues of curcumin to improve the drawbacks of its clinical application (Tagde et al., 2021; Rafiee et al., 2019).

**FIGURE 1:**
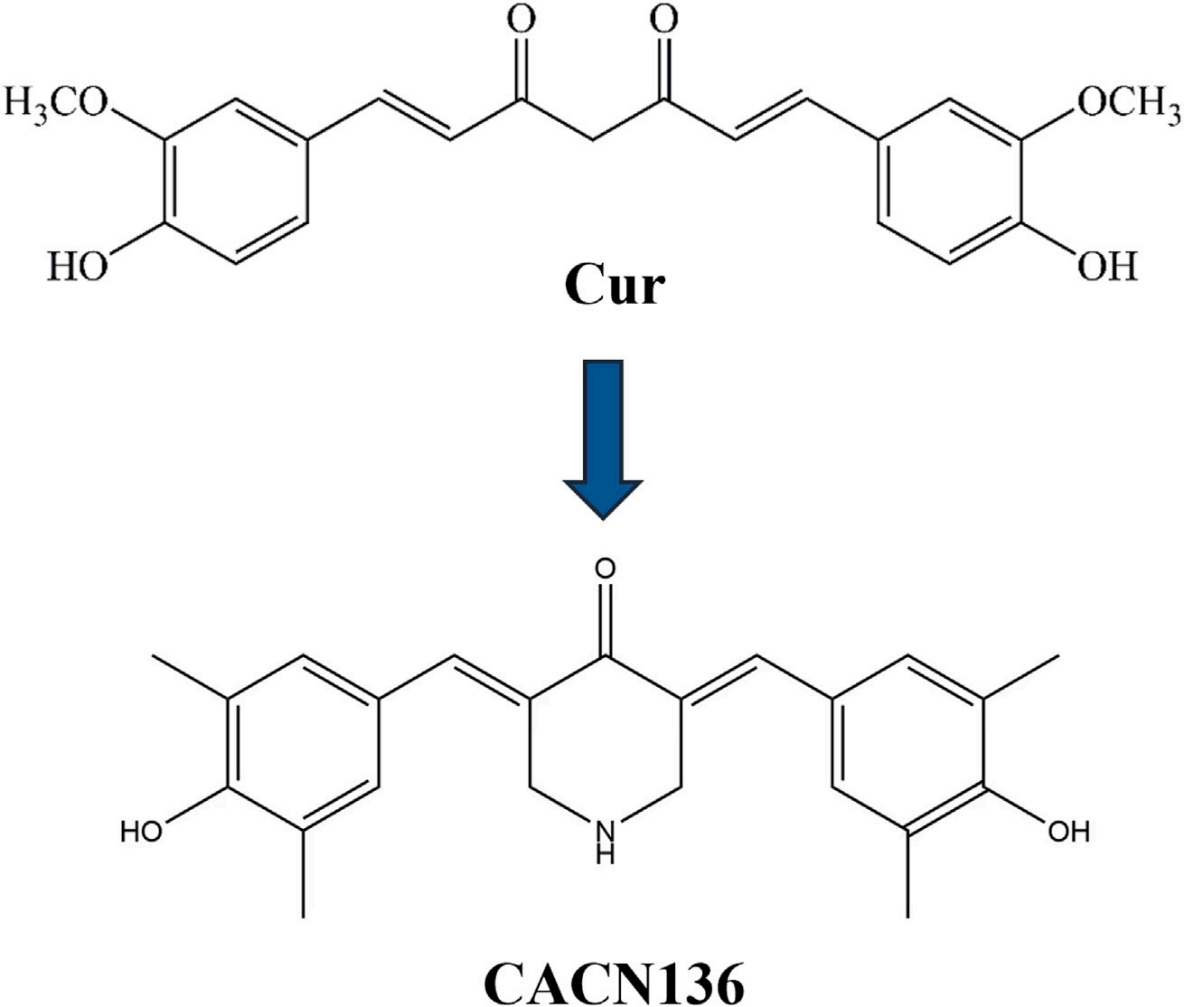
CACN136 is a structural analog of curcumin.

Therefore, a series of curcumin analogues were screened and synthesized by our group in previous studies (Zhao et al., 2021-09). Among them, a novel methyl monocarbonyl curcumin analogue named CACN136 (Figure 1) showed significant antidepressant *in vitro* and *in vivo* (Zhao et al., 2021-09), which was able to show significant protection against oxidative stress injured cells at all concentrations, and compared with ascorbic acid and curcumin, the CACN136 possesses a stronger 2,2′-Azinobis-(3-ethylbenzthiazoline-6-sulphonate) (ABTS) free radical ion scavenging ability (Zhao et al., 2021-09; Chen et al., 2024). Whereas the high presence of free radicals leading to oxidative stress imbalance is one of the reasons for accelerating the early onset of atherosclerosis. It has also been shown that oxygen free radical is an important mediator in inflammation. A number of scholars have found that there is a causal relationship between depression and atherosclerosis co-morbidity (Babic et al., 2023; Bezna et al., 2022; Neil et al., 2017). Meanwhile, CACN136 had a higher solubility (150.4 μg/mL) than curcumin, suggesting the possibility of better *in vivo* absorption (Wen et al., 2024). Our research team also found that orally administering CACN136 to rats fed high-fat diets has a significant lipid-lowering effect. Based on our previous experimental results, we speculate that CACN136, as a novel curcumin analogue, has a stronger anti-atherosclerosis effect compared to curcumin.

This study aimed to systematically investigate the atherosclerosis effects and mechanisms of CACN136 to provide a theoretical basis and evidence support for developing novel atherosclerosis therapeutic agents. Firstly, an atherosclerosis model was established in high-fat diet-induced ApoE−/− mice, and CACN136 was confirmed to inhibit the formation of atherosclerosis plaques in mice using Oil Red O staining. Then, network pharmacology and transcriptomics were utilized to predict the mechanism of action and targets of CACN136. *In vitro*, the ability of CACN136 to regulate polarization and lipid metabolism was assessed by the LPS-induced RAW264.7 cell polarization model and oxidized-LDL-induced RAW264.7 foam cell model, respectively. *In vivo*, Elisa assay and immunohistochemistry were performed to assess the *in vivo* relevant mechanisms and targets of CACN136. The experimental results suggest that CACN136 is expected to be a new drug candidate for the treatment of atherosclerosis.

## 2 Methods and materials

### 2.1 Evaluation of the anti-atherosclerosis effect of CACN136 *in vivo*

#### 2.1.1 Animals

Male ApoE knock-out mice (ApoE−/−, 6 weeks) on the C57BL/6 background were purchased from Suzhou Saiye Biotechnology Co., Ltd (Suzhou, China). The mice were housed in a specific pathogen-free facility under controlled conditions (temperature, 22°C ± 2°C; relative humidity, 55% ± 15%; noise, <60 dB; light/dark cycle, 12/12 h). Male ApoE−/− mice were fed with a high-fat diet (HFD) for 12 weeks to induce atherosclerosis. C57BL/6 mice served as the control group, while ApoE−/− mice induced by HFD were randomly divided into 5 groups with 6 mice in each group. The groups were as follows: model group (Model), CACN136 10 mg/kg group (Low), CACN136 20 mg/kg group (Middle), CACN136 40 mg/kg group (High), Sim 10 mg/kg group (Sim). The mice were orally gavaged with the respective treatments at a fixed time every day for 28 days. The model group mice were orally gavaged with an equal volume of saline. The mice were weighed once a week at a fixed time, and their body weights were recorded. All animal experiments were conducted in accordance with the ethical guidelines for animal welfare and approved by the ethical review board of Southwest Medical University, with the approval number: 20221116-022.

#### 2.1.2 Atherosclerosis animal model establishment and evaluation

After the adaptation period, C57BL/6 mice continued to have free access to regular diet, and the ApoE−/− mice were switched to a high-fat diet (HFD) for 12 weeks (Zhou et al., 2017). The mice were weighed once a week at a fixed time, and their body weights were recorded.

The mice were fixed in a supine position on a metal electrode on an ultrasound table, with their nose and mouth placed in a mask continuously delivering isoflurane anesthesia. The operating table was maintained at a constant temperature of 37°C. The MS-400 probe of Vevo 2,100 (VisualSonics Inc., Canada) was used to obtain images of the aortic arch section and the parasternal short-axis section next to the sternum. Doppler pulse images of the descending aorta were collected in the aortic arch section, and B-mode images of the aortic arch and its branches were obtained. M-mode images were obtained in the parasternal short-axis section, allowing measurement or calculation of left ventricular anterior wall thickness in diastole and systole (LVAWd, LVAWs), left ventricular posterior wall thickness in diastole and systole (LVPWd, LVPWs), left ventricular internal diameter in diastole and systole (LVIDd, LVIDs), and interventricular septal thickness in diastole and systole (IVSd, IVSs).

#### 2.1.3 CACN136 *in vivo* safety evaluation

After the treatment period, all animals were euthanized by decapitation after blood collection from the orbital sinus. The collected blood from the orbital sinus was collected in centrifuge tubes coated with EDTA and centrifuged at 5,000 rpm for 15 min. The levels of aspartate aminotransferase (AST) and alanine aminotransferase (ALT) in the mouse plasma samples were measured using a veterinary biochemical analyzer (BS-240VET).

H&E staining was performed on paraffin sections of various organ tissues and aortic tissue from the mice.

#### 2.1.4 Oil red O staining in en face and aortic root of apoE−/− mice

The fixed intact aorta was removed from the paraformaldehyde and placed in PBS. The aorta was finely trimmed under a somatic microscope to remove as much excess connective tissue and peripheral fat around it as possible. Subsequently, the trimmed aorta was stained by immersion in oil red O stain for 30 min and washed three times with 70% alcohol for 5 min each time after completion of staining to remove excess stain. Next, the spread aorta was cut along the midline with microscopic scissors to fully expose the inner wall of the aorta.

The aortic root sample was removed from the −80°C refrigerator and trimmed to a length of approximately 2 mm. A layer of OCT embedding adhesive was applied to the sample tray, the trimmed tissue was placed on it, and then covered with OCT adhesive to completely encapsulate the tissue, which was then placed into a thermostatic frozen slicer for freezing and solidification. After the samples were solidified, they were serially sectioned with a thickness of 5–6 μm using a thermostatic freezer sectioning machine. After sectioning, the following steps were performed sequentially: formaldehyde fixation for 10 min, distilled water washing for 3 times (10 min each time), staining with oil-red O staining solution for 10 min, and then distilled water washing for 3 times (10 min each time), hematoxylin re-staining, hydrochloric acid-ethanol differentiation for 2–3 s, tap water rinsing (bluing) for 1–3 min, distilled water washing for 1–3 min, and then staining with OCT staining solution. −3 min, distilled water washing 3 times (10 min each), ammonia reblueing for 3–4 s, and finally distilled water washing 3 times (10 min each). The slices were dried slightly and then sealed with glycerol. Image acquisition of the sections was performed using a BA210Digital trinocular camera microscope camera system manufactured by McAudi Industrial Group Ltd.

The red areas were considered as atherosclerotic lesions, and the lesion area (%) was calculated using ImageJ software:

Lesion area (%)=(Red area)/(Total area of the aorta)×100%

### 2.2 Prediction of the mechanism of action of CACN136 anti-AS

#### 2.2.1 Network pharmacology

Network pharmacology Disgenet database (https://www.disgenet.org/), Genecards database (https://www.genecards.org/), and OMIM database (https://www.omim.org/) were used to identify target genes related to atherosclerosis disease. Swiss Target Prediction (http://www.swisstargetprediction.ch) was employed to predict the target proteins of CACN136 (Synthesized by the Pharmacy Laboratory of Southwest Medical University with independent intellectual property rights, purity >99%). The intersection of disease-drug targets was determined. The intersection targets were then input into the String database (https://string-db.org/) to construct protein-protein interaction networks. Enrichment analysis of KEGG pathways and GO functions for the intersection targets was performed using the David database (https://david.ncifcrf.gov/).

#### 2.2.2 Transcriptomics

RNA extraction was performed using TRIzol reagent (Invitrogen, cat. NO 15596026) following the method described by Chomczynski et al. (Chomczynski and Sacchi, 1987). The three groups of samples (blank group, LPS model group, drug group) were subjected to RNA extraction. The KC-DigitalTM Stranded mRNA Library Prep Kit for Illumina^®^ (Catalog NO. DR08502, Wuhan Seqhealth Co., Ltd. China) was used for chain-specific RNA sequencing library preparation according to the manufacturer’s instructions. Finally, PE150 sequencing was performed in the DNBSEQ-T7 sequencer (MGI Tech Co., Ltd. China). Differential gene expression was determined using the DESeq R package. Differential genes with Log2|Fold Change|≥0.58 between the LPS model group and the blank group, as well as between the LPS model group and the drug group, were selected. The intersection of the two sets of differential genes was obtained. The obtained intersection targets were subjected to topological analysis using Cytoscape software to identify core targets. Finally, KEGG pathway enrichment analysis was performed using the David website.

### 2.3 Investigating the mechanism of CACN136 anti-atherosclerosis action *in vitro*

#### 2.3.1 Cell culture and grouping

The RAW264.7 macrophage cell line derived from mice was obtained from the Institute of CVD, Southwest Medical University. After resuscitation, RAW264.7 cells were transferred to DMEM high-glucose culture medium (Gbico) containing 10% fetal bovine serum (Zhejiang Tianhang Biological Technology Co., Ltd.) and 2% penicillin-streptomycin solution (Shanghai Biyuntian Biotechnology Co., Ltd.). The cells were cultured in a cell incubator at a temperature of 37°C and 5% CO_2_. Polarization model: Well-growing RAW264.7 cells were evenly seeded in various wells of a plate. After 4 h of incubation, the cells adhered to the bottom. The cells were randomly divided into Control group, LPS group (1 μg/mL), CACN136 (0.6 μg/mL, 0.3 μg/mL, 0.15 μg/mL) + LPS (1 μg/mL) group. Foam cell model: Well-growing RAW264.7 cells were evenly seeded in various wells of a plate. After 4 h of incubation, the cells adhered to the bottom. The cells were then starved in serum-free medium for 6 h and randomly divided into Control group, ox-LDL group (60 μg/mL), CACN136 (0.6 μg/mL, 0.3 μg/mL, 0.15 μg/mL) + ox-LDL (60 μg/mL) group.

#### 2.3.2 Cell cytotoxicity assay

The impact of the drug on cell proliferation viability was determined using the MTT colorimetric assay, as previously described (Wei et al., 2022). The drug concentrations were set as a gradient of 4.8 μg/mL, 2.4 μg/mL, 1.2 μg/mL, 0.6 μg/mL, 0.3 μg/mL, and 0.15 μg/mL.

#### 2.3.3 Q-PCR experiment

Total RNA was extracted from cell samples using the FastPure Cell/Tissue Total RNA Isolation Kit V2 (Nanjing NuoWeiZan Biotech Co., Ltd.). HiScript III Qrt SuperMix (Nanjing NuoWeiZan Biotech Co., Ltd.) was used for reverse transcription. ChamQ Universal SYBR qPCR Master Mix (Nanjing NuoWeiZan Biotech Co., Ltd.) was used for mRNA level detection. The primer sequences (Shanghai Bioengineering Co., Ltd.) are shown in Table 1.

**TABLE 1:** Primer sequences.

Gene	Primer sequence 5′-3′,F	Primer sequence 5′-3′,R
IL-1β	ACC​CCA​AAA​GAT​GAA​GGG​CTG​CTT	TGC​CTG​CCT​GAA​GCT​CTT​GTT​GAT
iNOS	CTT​GGA​GCG​AGT​TGT​GGA​TTG	GGT​CGT​AAT​GTC​CAG​GAA​GTA​GGT
TGF-β	GCG​GAC​TAC​TAT​GCT​AAA​GAG​G	CAC​TGC​TTC​CCG​AAT​GTC​T
Arg1	GGAGAAGGCGTTTGCTTAGTTC	GGAGAAGGCGTTTGCTTAGTTC
SR-B1	TTTCAGCAGGATCCATCTGGTGGA	AGTTCATGGGGATCCCAGTGAC
ABCA1	GGAGCTGGGAAGTCAACAAC	ACATGCTCTCTTCCCGTCAG
CD36	GGAGCCATCTTTGAGCCTTCA	GAACCAAACTGAGGAATGGATCT
SRA1	TTCACTGGATGCAATCTC	CTTGGCTTGCTTCGGAACTC

#### 2.3.4 Western blot experiment

After collecting and washing the cell samples, they were dissolved in RIPA buffer. The protein concentration was measured using the BCA assay kit. The proteins were separated by SDS-PAGE electrophoresis using an appropriate concentration and then transferred onto a polyvinylidene fluoride (PVDF) membrane. After blocking with the blocking buffer, the membranes were incubated overnight at 4°C with antibodies against iNOS(80517-1-RR, Proteintech, China), Arg1 (66129-1-Ig, Proteintech, China), ABCA1(66217, Abcam, United States), ABCG1 (13578-1-AP, Proteintech, China), CD36 (18836-1-AP, Proteintech, China), and SRA1 (24655-1-AP, Proteintech, China). Then, the membranes were incubated with a secondary antibody (goat anti-rabbit IgG H&L (HRP) at room temperature for 30 min with shaking. Tubulin antibody (66031-1-Ig, Proteintech, China) was used as an internal control, and ImageJ software was used for protein band densitometry analysis.

#### 2.3.5 Oil red O staining experiment

After washing twice with pre-chilled PBS buffer, the cells were fixed in Cell ORO Fixative at room temperature for 15–25 min in the dark, followed by washing with 60% isopropanol. The cells were then stained with Oil Red O staining solution in a dark room at 37°C for 15 min, followed by washing with 60% isopropanol and rinsing with ultrapure water for 10 min. After drying, the cells were observed and photographed under a microscope.

#### 2.3.6 Cholesterol detection experiment

Cholesterol quantification was performed using the total cholesterol assay kit (BC1985, Solarbio, China) and the free cholesterol assay kit (BC1895, Solarbio, China), following the manufacturer’s instructions.

#### 2.3.7 Dil-ox-LDL uptake experiment

Following the manufacturer’s instructions, 20 μg/mL Dil-ox-LDL (YB-0010, Yiyuan, China) was added to each well containing cells on coverslips in the dark. After 4 h of cell uptake, the coverslips were removed, stained with DAPI dye (KGF0218, Keygen, China), and coverslipped. The cells were then observed and photographed using a fluorescence inverted microscope.

#### 2.3.8 Cholesterol efflux function assay

Following the manufacturer’s instructions, while treating the cells with the drug, each group of cells was simultaneously treated with 5 μg/mL of 25-NBD-cholesterol. After 24 h of incubation, the culture medium was discarded, and each group of cells was exchanged with serum-free DMEM medium containing 50 μg/mL HDL. The cells were further incubated for 4 h, and the supernatant from each well was collected into a 96-well plate. Blank medium was added to the original cell wells in the same volume as the supernatant wells. The fluorescence intensity was measured using a microplate reader with an excitation wavelength of 485 nm and an emission wavelength of 535 nm. The cholesterol efflux rate (%) was calculated using the following formula:

Cholesterol efflux rate (%) = $\frac{\text{Fluorescence}\;\text{intensity}\left(supernatant\right)}{\text{Total}\;\text{fluorescence}\;\text{intensity}\left(supernatant+cells\right)}$ × 100%

### 2.4 Investigating the mechanism of CACN136 anti-AS action *in vivo*

#### 2.4.1 Plasma cholesterol level measurement

Total cholesterol (TC), triglycerides (TG), low-density lipoprotein cholesterol (LDL-C), and high-density lipoprotein cholesterol (HDL-C) levels in mouse plasma samples were measured using a veterinary biochemical analyzer (BS-240VET).

#### 2.4.2 Elisa assay

According to the manufacturer’s instructions, corresponding levels of inflammatory factors TNF-α and IL-6 in mouse plasma were detected using Elisa kits (920, 14206, Meimian, China).

#### 2.4.3 Immunohistochemistry

First, paraffin-embedded sections of mouse aortic tissue were deparaffinized and rehydrated. The sections were then boiled in 0.01 M sodium citrate buffer (pH 6.0) for 10 min (temperature maintained above 95°C) for antigen retrieval. Next, endogenous peroxidase was blocked using 3% hydrogen peroxide, and after room temperature blocking, the sections were incubated overnight at 4°C with primary antibodies against iNOS, Arg1, ABCG1, and CD36 (1:500). Finally, the sections were incubated with secondary antibody reagents for 30 min at room temperature.

### 2.5 Statistical analysis

The data are presented as mean ± SEM (standard error of the mean). Statistical significance was evaluated using one-way analysis of variance (ANOVA) with SPSS 22.0 and GraphPad Prism 5.0 for multiple group comparisons. P < 0.05 indicates statistical significance.

## 3 Results

### 3.1 The anti-atherosclerosis effect of CACN136

#### 3.1.1 Successful construction of atherosclerosis model mice

**FIGURE 2:**
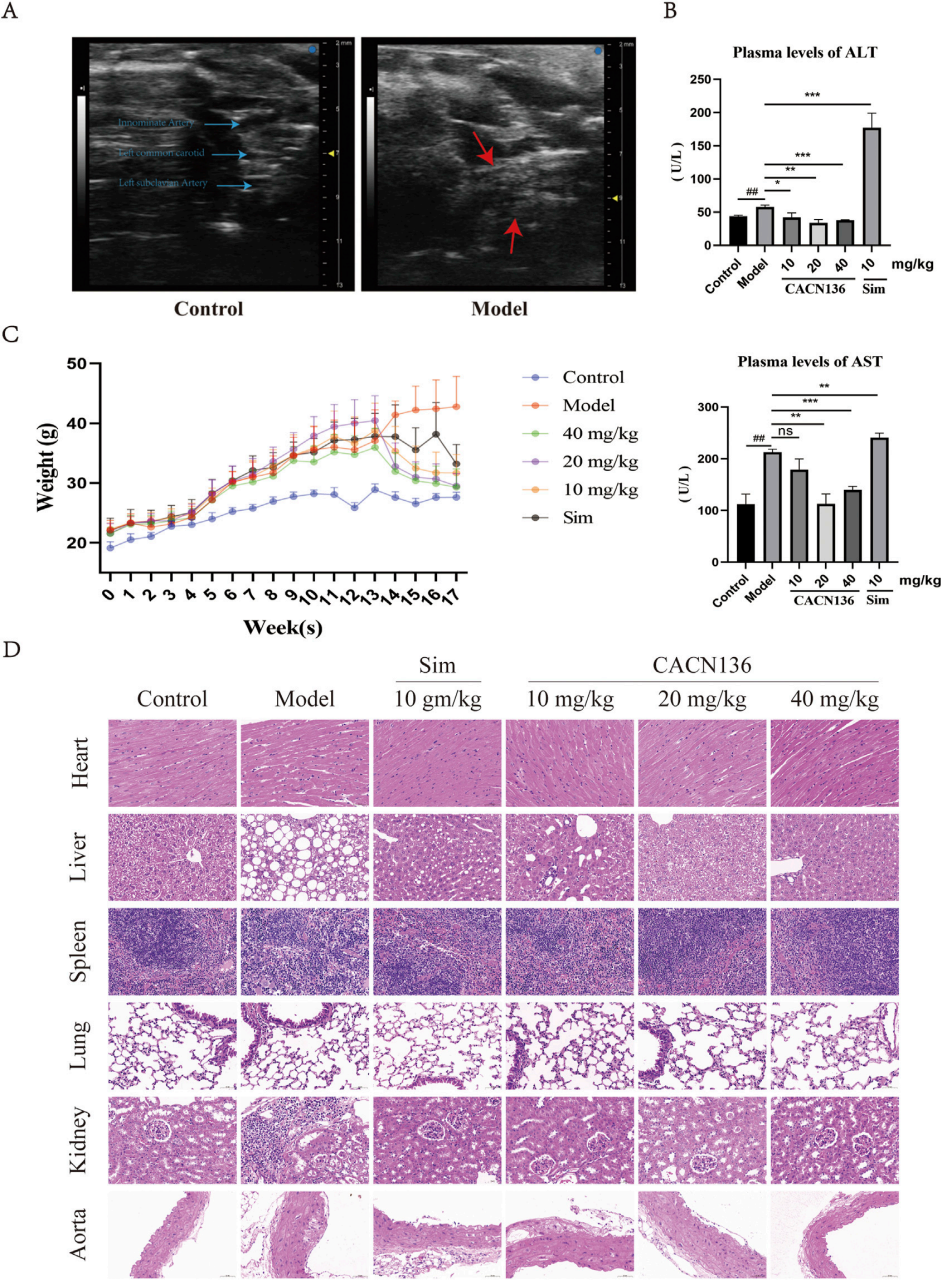
CACN136 has good safety. **(A)** Mice aortic arch ultrasound B-Mode image; **(B)** Plasma ALT and AST levels in mice ( $\bar{x}$ ± s, n = 3).^##^P < 0.01, compared with the Control group.*P < 0.05, **P < 0.01, ***P < 0.001, compared with the model group. **(C)** mouse body weight curve ( $\bar{x}$ ± s, n = 6); **(D)** HE staining of mice organs (100×).

Mouse atherosclerosis modeling results was evaluated using Ultrasound Imaging System. After 12 weeks of modeling, the control group mice exhibited clear boundaries, smooth vessel walls, and uniform thickness in the aortic arch and its three branches (from top to bottom: innominate artery, left carotid artery, left subclavian artery). In contrast, the model group mice showed significant dilation of the aortic arch, with a large area of plaque formation at the junction with the left carotid artery. The innominate artery exhibited wall dilation and the presence of calcified lesions, while the outlines of the left carotid artery and left subclavian artery were blurred and almost completely covered by plaques (Figure 2A). These findings indicate the successful establishment of the atherosclerosis mouse model. Compared to the control group mice, the atherosclerosis model mice exhibited a significant decrease in ejection fraction and fractional shortening of 10.63% (p < 0.05) and 9.43% (p < 0.05), respectively (Table 2). The left ventricular corrected mass significantly increased by 30.92 mg (p < 0.05), and the aortic blood flow velocity decreased significantly by 65.70% (p < 0.01) (Table 2). These results indicate a significant decrease in cardiac function, increased cardiac burden, and gradual transition to myocardial hypertrophy in the model mice. The mouse growth curve (Figure 2C) revealed that during the modeling period, all mice’s body weight gradually increased. However, during the treatment period, the body weight of all dosing groups continued to decrease in a concentration-dependent manner. At the end of the treatment, the weight loss effect of all CACN136 groups was slightly better than that of the positive control group treated with Sim.

**TABLE 2:** Cardiac ultrasound related indexes of mice ( $\bar{x}$ ± s, n = 3).

Indexes	Units	Control	Model
EF	%	77.62 ± 8.16	66.99 ± 6.67*
SF	%	45.51 ± 7.74	36.08 ± 5.16*
LVM	mg	113.80 ± 22.06	186.68 ± 92.70
LVCM	mg	91.04 ± 17.65	121.96 ± 17.22*
Flow velocity	mm/s	892.42 ± 118.95	586.32 ± 106.76***
BP	mmHg	3.23 ± 0.82	1.41 ± 0.51**

#### 3.1.2 CACN136 safety is good

Due to the first-time use of CACN136 for treating ApoE−/− mice, the liver function was evaluated by measuring the blood biochemical indicators, and the organ safety was assessed using HE staining. As shown in Figure 2B, compared to the model group, the ALT and AST levels were significantly decreased in the blank group and treatment group, while the positive drug Sim group showed a significant increase compared to the model group, with an ALT value exceeding the normal range (28–132 U/L) reaching 177.2 ± 17.78 U/L. The HE staining results of the mouse organs in each group are shown in Figure 2D. The blank group and CACN136 high-dose group showed no significant pathological changes in any organs. However, the model group exhibited disrupted liver tissue structure, diffuse fatty degeneration of liver cells with abundant lipid droplets in the cytoplasm, and localized plaque formation in the aorta tissue. The CACN136 medium-dose group showed mild vacuolar degeneration in liver cells, and a small amount of plaque was observed in the aorta tissue. The low-dose group showed mild vacuolar degeneration in liver cells, and a small amount of plaque was observed in localized areas of the aorta tissue. The Sim group showed slight fatty degeneration in liver cells and extremely few plaques were observed in the aorta tissue.

#### 3.1.3 CACN136 alleviated atherosclerosis lesions in HFD-induced ApoE−/− mice

**FIGURE 3:**
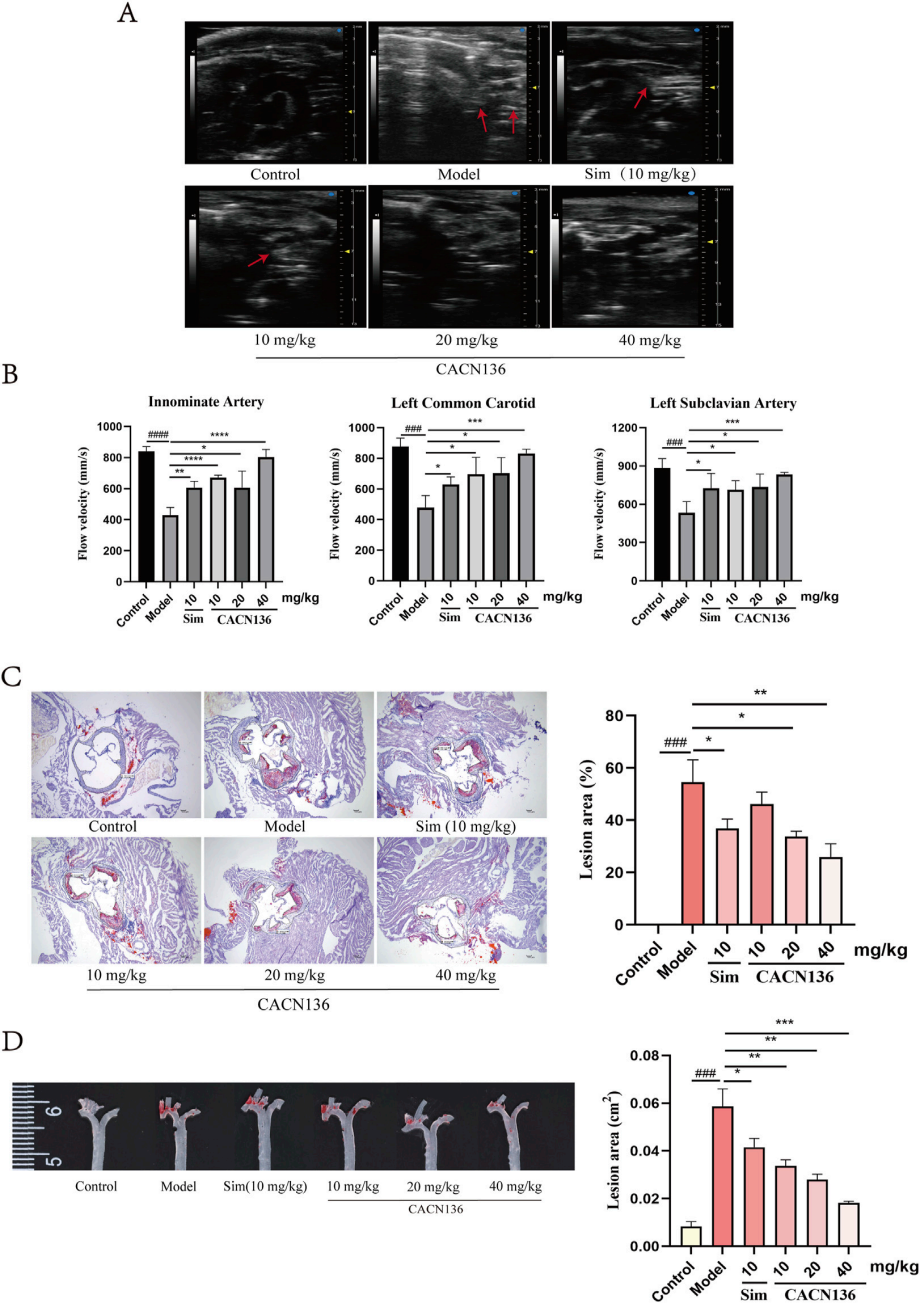
CACN136 alleviated atherosclerosis lesions in HFD-induced ApoE−/− mice. **(A)** Mice aortic arch ultrasound B-Mode image; **(B)** Blood flow velocity (mm/s) in the innominate artery, left common carotid artery, and left subclavian artery of mice ( $\bar{x}$ ± s, n = 3); **(C)** Oil red O staining of mice aortic root and Quantification of oil red O staining of mice aortic root ( $\bar{x}$ ± s, n = 3); **(D)** Oil red O staining of the entire aorta and Quantification of oil red O staining of the entire aortic ( $\bar{x}$ ± s, n = 3). ###P < 0.001, compared with the Control group.*P < 0.05, **P < 0.01, ***P < 0.001, ****P < 0.0001, compared with the model group.

The ability of CACN136 to alleviate atherosclerosis lesions was evaluated using Ultrasound Imaging System and Oil Red O Staining. As shown in Figure 3A, the blank group of mice had no atherosclerosis lesions in the aortic arch and its three branches. In contrast, the model group of mice exhibited large plaque areas in the aortic arch, calcification lesions in the arterial walls of the branch arteries, indistinct contours of the innominate and left carotid arteries almost completely covered by plaques, and dilatation of the left subclavian artery. The positive drug Sim group and the CACN136 treatment group showed clearer contours of the aortic arch compared to the model group, with significantly reduced plaque formation. In particular, the high-dose and medium-dose groups exhibited significant improvement in arterial wall calcification, while the positive drug and low-dose groups showed more calcification in the innominate artery wall. As shown in Figure 3B, the blood flow velocity in the three branches of the model group was significantly lower compared to the blank group of mice. However, in all treatment groups, the blood flow velocity in the three branches significantly increased compared to the model group, indicating that CACN136 can reduce plaque formation and improve blood flow velocity, bringing it closer to normal levels. Figure 3C shows the cross-section of the aorta stained with oil-red O. The positive area in the model group reached 54.63% ± 6.91%. The positive areas in the treatment groups, from high to low, were as follows: CACN136 low-dose group (46.13% ± 3.78%), Sim group (36.83% ± 2.95%), CACN136 medium-dose group (33.74% ± 1.68%), and CACN136 high-dose group (25.92% ± 4.09%). Figure 3D shows the entire aorta stained with oil-red O. The positive area in the model group reached 0.059 ± 0.006 cm^2^. The positive areas in the treatment groups, from high to low, were as follows: Sim group (0.041 ± 0.003 cm^2^), CACN136 low-dose group (0.0034 ± 0.002 cm^2^), CACN136 medium-dose group (0.028 ± 0.002 cm^2^), and CACN136 high-dose group (0.018 ± 0.001 cm^2^).

### 3.2 Prediction of the anti-atherosclerosis mechanism of CACN136

#### 3.2.1 Network pharmacology analysis

**FIGURE 4:**
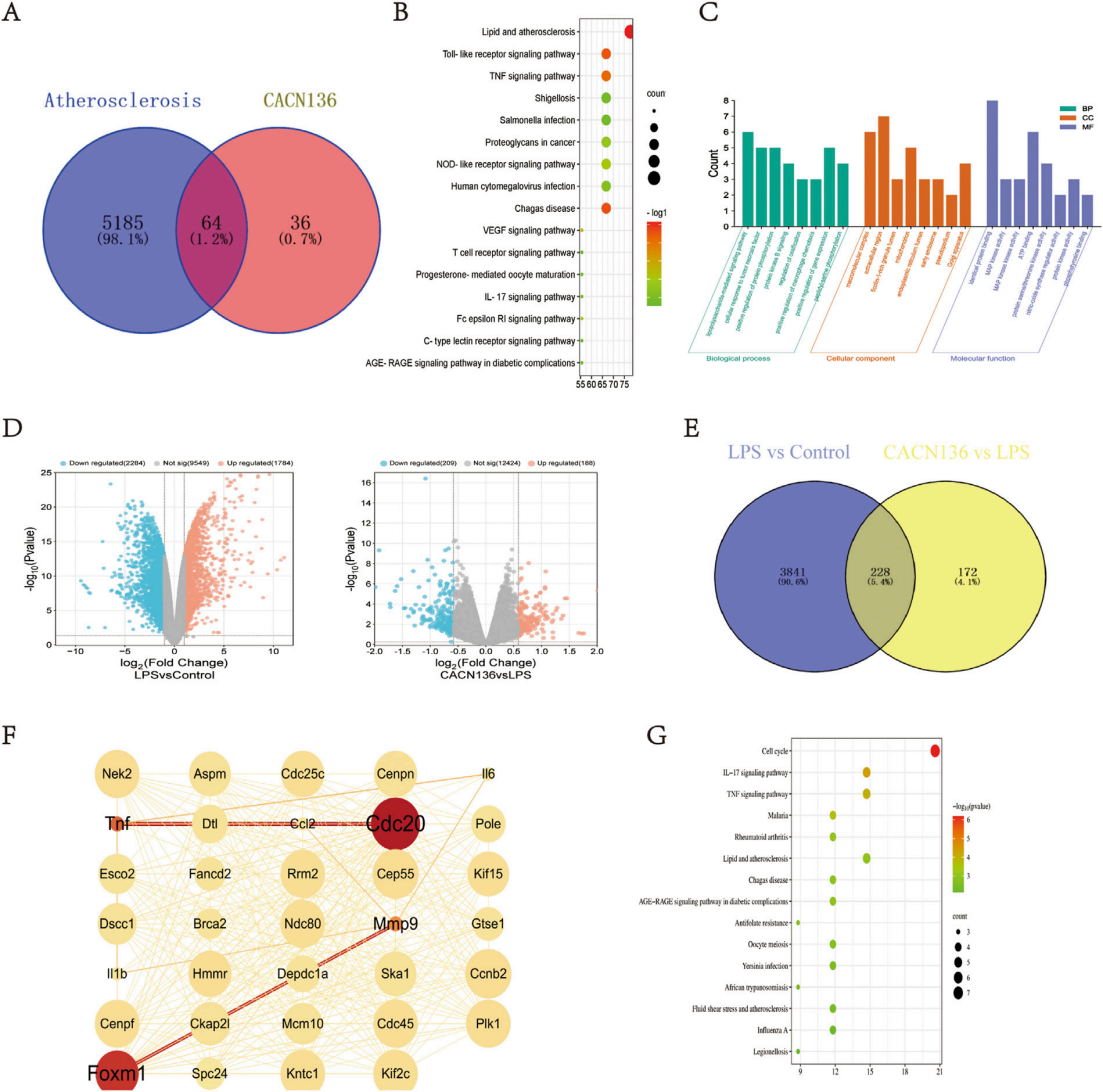
CACN136 inhibits atherosclerosis by regulating lipid metabolism and inflammation-related pathways. **(A)** Disease-drug target veen plots; **(B)** Network pharmacology KEGG enrichment plots; **(C)** Network pharmacology GO enrichment plots; **(D)**. Transcriptome differential gene volcano plots; **(E)** LPS vs. Control vs. CACN136 vs. LPS differential gene veen plots; **(F)** Differential gene core targets; **(G)** Transcriptome differential gene KEGG enrichment plots.

To predict the mechanism of CACN136 anti-atherosclerosis, a total of 5,249 target genes related to atherosclerosis were obtained from the OMIM, Genecards, and DisGeNET databases. Additionally, 100 potential target genes of CACN136 were predicted using the SwissTargetPrediction database. The intersection of the predicted atherosclerosis target genes and CACN136 target genes resulted in 64 common target genes (Figure 4A). The KEGG enrichment analysis results (Figure 4B) showed that CACN136 had the most significant enrichment effect on lipid metabolism and atherosclerosis pathways. The enriched pathways included lipid metabolism and atherosclerosis pathway, Toll-like receptor signaling pathway, TNF signaling pathway, NOD-like receptor signaling pathway, vascular endothelial growth factor (VEGF) signaling pathway, T cell signaling pathway, IL-17 signaling pathway, C-type lectin receptor signaling pathway, and AGE-RAGE signaling pathway in diabetic complications. The GO enrichment analysis results (Figure 4C) showed that the mechanisms by which CACN136 exerts its anti-atherosclerosis effects are mainly involved in biological processes such as LPS-mediated signaling pathway, cellular response to tumor necrosis factor, positive regulation of protein phosphorylation, positive regulation of macrophage chemotaxis, positive regulation of gene expression, and molecular functions such as MAP kinase activity, ATP binding, protein serine/threonine kinase activity, nitric oxide synthase regulator activity, protein kinase activity, and tyrosine phosphorylation binding.

#### 3.2.2 Transcriptomic analysis of differentially expressed genes

Using RNA-seq, the differentially expressed genes between macrophage communities after treatment with CACN136 were investigated. A total of 4,068 differential genes (1784 upregulated and 2,284 downregulated) were observed in the LPS group relative to the model group, as shown by the volcano plot visualization. Similarly, in the CACN136 group relative to the LPS group, 397 differential genes (188 upregulated and 209 downregulated) were identified (Figure 4D). A total of 228 target genes were obtained by plotting the Venn diagram to take the intersection (Figure 4E). A protein-protein interaction (PPI) network was further constructed and topological analysis was performed using Cytoscape, identifying 34 core target genes (Figure 4F). KEGG enrichment analysis using David (Figure 4G) revealed that these enriched pathways mainly included the IL-17 signaling pathway, TNF signaling pathway, lipid and atherosclerosis signaling pathway, and fluid shear stress signaling pathway.

### 3.3 CACN136 regulates macrophage polarization and lipid metabolism *in vitro*

#### 3.3.1 Regulation of LPS-induced macrophage RAW264.7 M1/M2 polarization by CACN136

**FIGURE 5:**
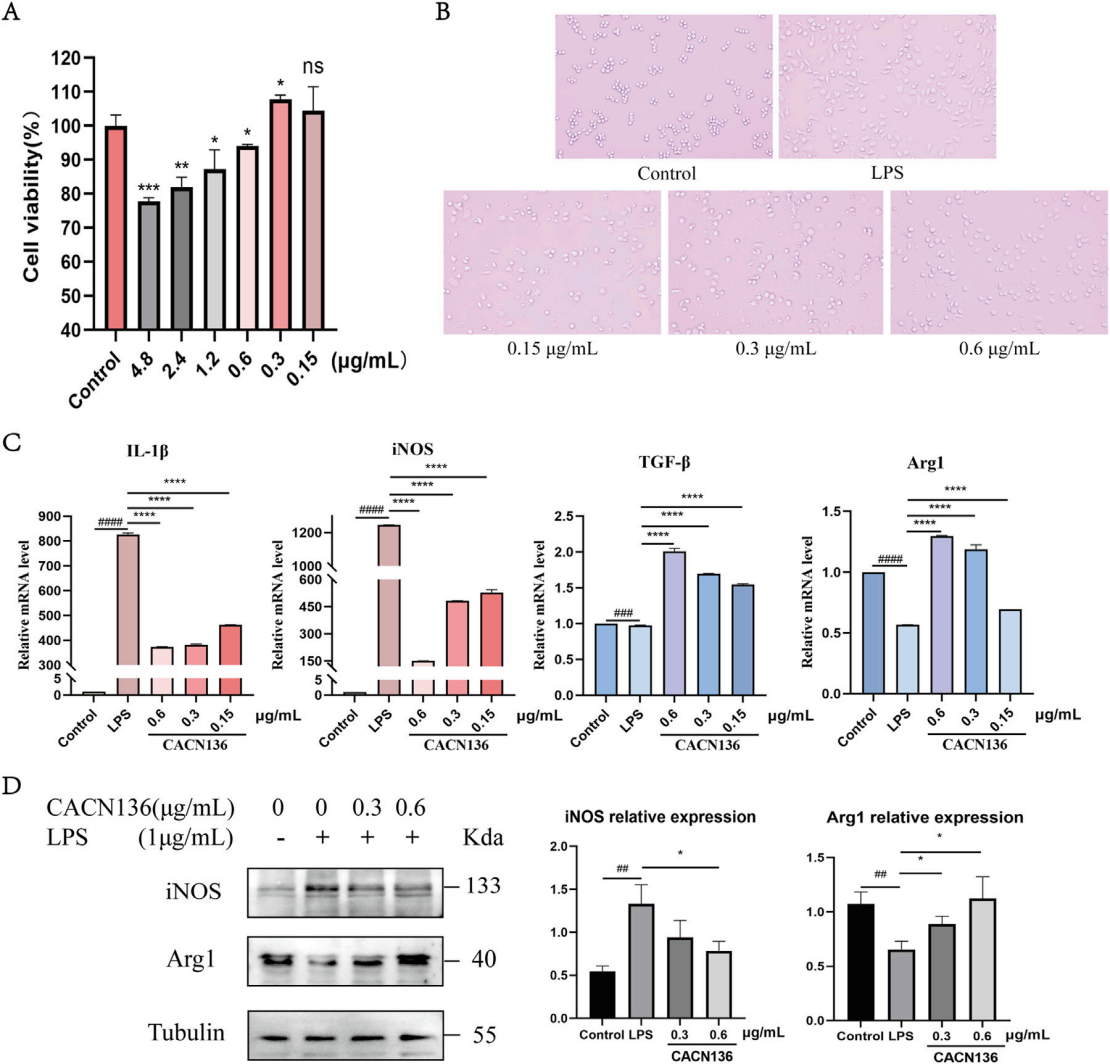
CACN136 regulates macrophage polarization through the iNOS/Arg1 axis. **(A)** Effect of CACN136 intervention for 24h on RAW264.7 viability ( $\bar{x}$ ± s, n = 3); **(B)** Morphological changes in RAW264.7 cells; **(C)** Effect of CACN136 on mRNA levels in RAW264.7 cells ( $\bar{x}$ ± s, n = 3); **(D)** Effect of CACN136 on protein levels in RAW264.7 cells ( $\bar{x}$ ± s, n = 3). ^##^P < 0.01,^###^P < 0.001, ^####^P < 0.0001, compared with the Control group. *P < 0.05, **P < 0.01,***P < 0.001, ****P < 0.0001, compared with the LPS model group.

The effect of CACN136 on the viability of RAW264.7 cells was evaluated using the MTT assay (Figure 5A). Based on the results, the administration concentrations of CACN136 for subsequent experiments were chosen to be 0.6, 0.3 and 0.15 μg/mL. Under the microscope, the morphology of RAW264.7 cells after LPS induction was observed (Figure 5B). The cells in the control group were mostly round or elliptical with high refractivity, appearing small and transparent. In the LPS group, most of the cells transformed into spindle shapes and extended elongated pseudopodia. The treatment groups with CACN136 showed a dose-dependent improvement in the irregular shape and number of pseudopodia in RAW264.7 cells. Subsequently, the mRNA expression levels of IL-1β, iNOS, TGF-β, and Arg1 in RAW264.7 macrophages were measured using q-PCR (Figure 5C). After 24 h of CACN136 intervention, the mRNA levels of IL-1β and iNOS showed a significant dose-dependent decrease (P < 0.0001). The high, medium, and low concentrations of CACN136 significantly increased the mRNA expression levels of TGF-β and Arg1, also in a dose-dependent manner. Furthermore, the results of Western blot experiments showed that the relative expression levels of iNOS protein in the CACN136 intervention group were significantly reduced to 0.59 times (0.6 μg/mL) and 0.7 times (0.3 μg/mL) compared to the model group (Figure 5D). At the same time, the relative expression levels of Arg1 protein in the CACN136 intervention group increased to 1.72 times (0.6 μg/mL) and 1.35 times (0.3 μg/mL) compared to the model group (Figure 5D).

#### 3.3.2 CACN136 inhibits ox-LDL-induced macrophage RAW264.7 transition to foam cells

**FIGURE 6:**
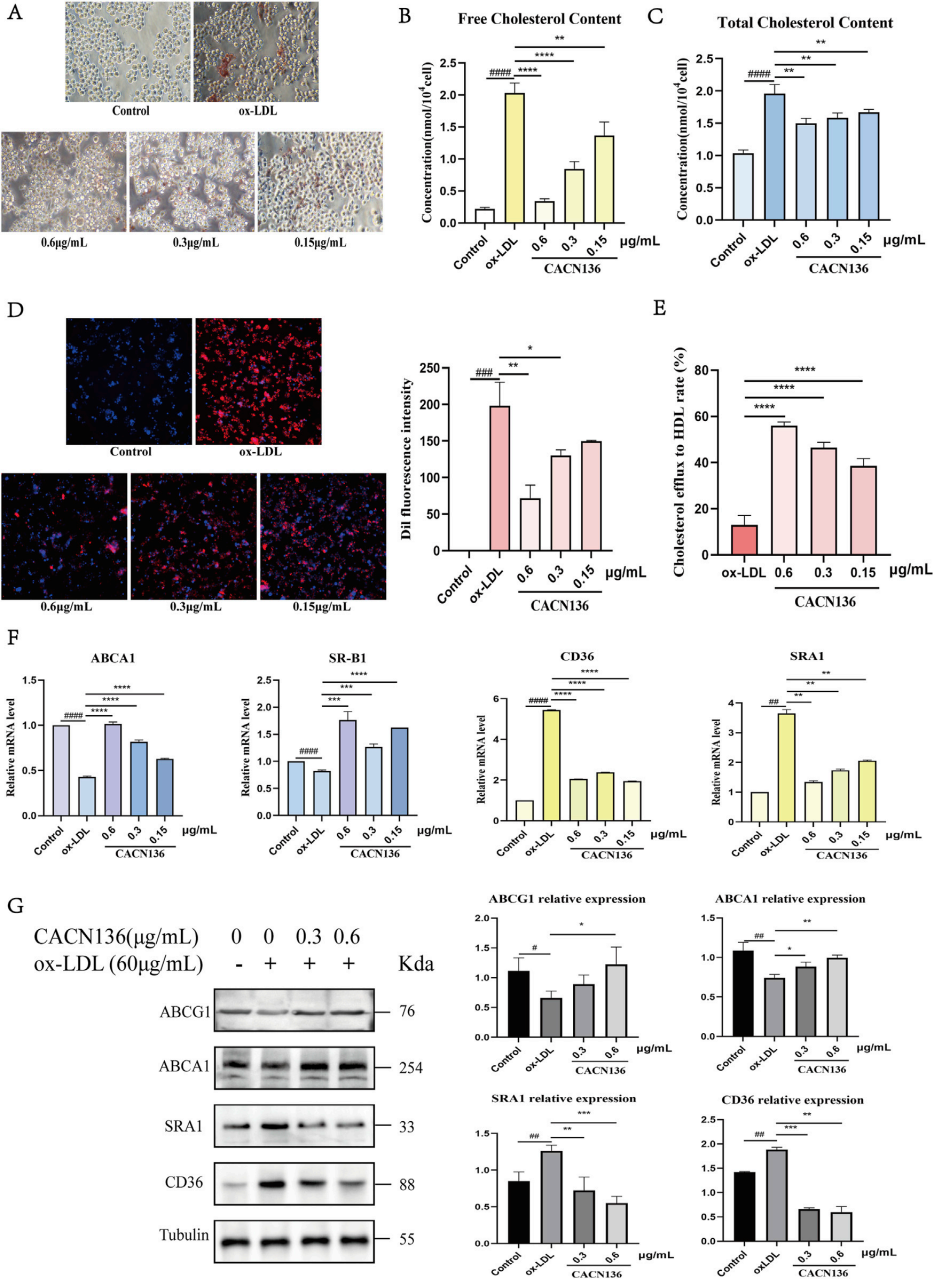
CACN136 modulates cholesterol metabolism pathways in foam cells. **(A)** Oil red O staining for detection of foam cell formation; **(B)** Free cholesterol content in RAW264.7 cells ( $\bar{x}$ ± s, n = 3); **(C)** Total cholesterol content in RAW264.7 cells ( $\bar{x}$ ± s, n = 3); **(D)** Ability of RAW264.7 cells to phagocytose Dil-ox-LDL (blue fluorescence for Dapi, red fluorescence for Dil) and Quantification of Dil fluorescence intensity ( $\bar{x}$ ± s, n = 3); **(E)** Cholesterol efflux rate ( $\bar{x}$ ± s, n = 3); **(F)** Effect of CACN136 on mRNA levels in RAW264.7 cells ( $\bar{x}$ ± s, n = 3); **(G)** Effect of CACN136 on protein levels in RAW264.7 cells ( $\bar{x}$ ± s, n = 3).^#^P < 0.05,^##^P < 0.01,^###^P < 0.001, ^####^P < 0.0001, compared with the Control group. *P < 0.05 **P < 0.01 ***P < 0.001, ****P < 0.0001, compared with the ox-LDL model group.

The formation of lipid droplets in cells after ox-LDL induction was observed using Oil Red O staining. (Figure 6A). The results showed that compared to the model group, the number of red lipid droplets in the cells of all CACN136 groups decreased, and the color became lighter. This indicates that CACN136 can inhibit the formation of macrophage-derived foam cells. Furthermore, the free cholesterol and total cholesterol levels in RAW264.7 cells treated with CACN136 were measured using assay kits. It was found that compared to the ox-LDL group, the levels of both free cholesterol (Figure 6B) and total cholesterol (Figure 6C) in the cells of all CACN136 groups were significantly reduced dose-dependent. Next, RAW264.7 cells were stained with Dil-ox-LDL fluorescent dye to observe the effect of CACN136 on the macrophage’s ability to engulf lipoproteins. The results (Figure 6D) showed that compared to the model group, the fluorescence intensity in the cells treated with CACN136 gradually weakened. This indicates that CACN136 can inhibit the uptake of ox-LDL by macrophages. In addition, RAW264.7 cells were intervened with NBD cholesterol to verify whether CACN136 could inhibit foam cell formation by promoting cholesterol efflux. The cholesterol efflux rate was calculated, and the results (Figure 6E) showed that the efflux rate in the model group was 12.95% ± 4.11%. After CACN136 intervention, the high, medium, and low dose groups increased the efflux rate by 4.32 times, 3.59 times, and 2.98 times, respectively.

The regulatory effect of CACN136 on lipid metabolism in RAW264.7 macrophages was also verified by measuring mRNA and protein expression levels associated with lipoprotein uptake and efflux. In terms of mRNA levels (Figure 6F) of ABCA1 and SR-B1, which control cholesterol efflux, and CD36 and SRA1, which control cholesterol uptake, compared to the control group, the model group showed a significant decrease in ABCA1 and SR-B1 mRNA levels (P < 0.0001) and a significant upregulation of CD36 and SRA1 mRNA levels (P < 0.01). After CACN136 intervention, the mRNA levels of ABCA1 showed a significant dose-dependent increase, and the mRNA levels of SR-B1 were significantly higher compared to the model group. CD36 and SRA1 are two specific receptors controlling cholesterol uptake. In the model group, the mRNA expression levels of CD36 and SRA1 were 5.44-fold and 3.65-fold higher than those in the control group, respectively. CACN136 significantly downregulated the mRNA levels of CD36 (P < 0.0001) and SRA1 (P < 0.01). At the protein level (Figure 6G), the high dose group showed a 1.88-fold increase in ABCG1 and a 4.28-fold increase in ABCA1 compared to the model group after CACN136 intervention. Meanwhile, CD36 and SRA1 were significantly downregulated.

### 3.4 CACN136 regulates inflammation and lipid metabolism *in vivo*

#### 3.4.1 CACN136 inhibited inflammation in HFD-induced ApoE−/− mice

**FIGURE 7:**
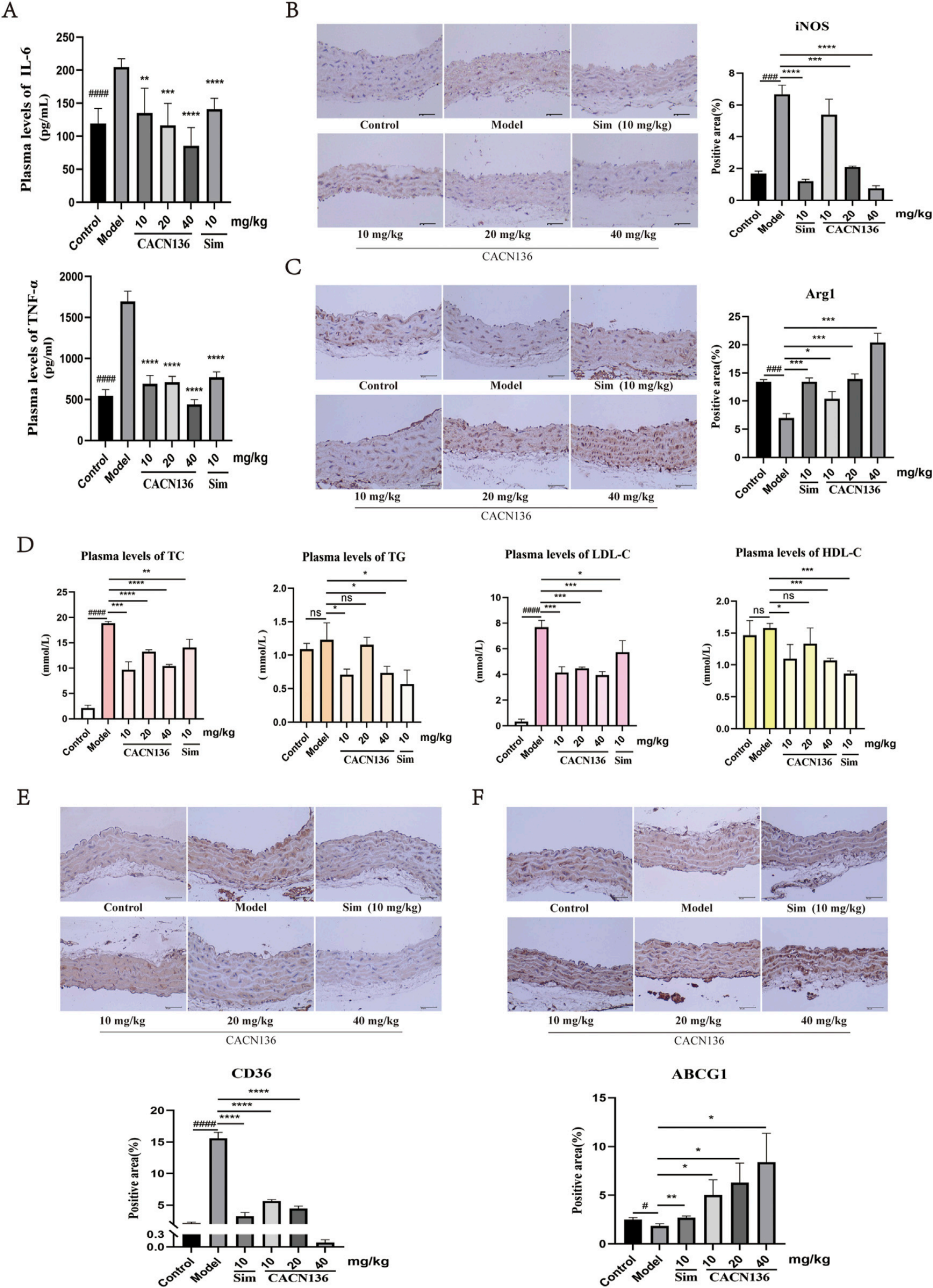
CACN136 improves inflammatory response and lipid metabolism in atherosclerotic mice. **(A)** Plasma levels of inflammatory factor TNF-α and IL-6 in mice ( $\bar{x}$ ± s, n = 3); **(B)** Aortic iNOS staining and Percentage area of positive aortic iNOS staining (%) ( $\bar{x}$ ± s, n = 3); **(C)** Aortic Arg1 staining and Percentage area of positive aortic Arg1 staining (%) ( $\bar{x}$ ± s, n = 3); **(D)** Plasma Cholesterol level assay in mice ( $\bar{x}$ ± s, n = 3); **(E)** Aortic CD36 staining and Percentage area of positive aortic CD36 staining (%) ( $\bar{x}$ ± s, n = 3); **(F)** Aortic ABCG1 staining and Percentage area of positive aortic ABCG1 staining (%) ( $\bar{x}$ ± s, n = 3).^#^P < 0.05,^###^P < 0.001, ^####^P < 0.0001, compared with the Control group.*P < 0.05, **P < 0.01, ***P < 0.001, ****P < 0.0001, compared with the model group.

HFD-induced atherosclerosis promotes inflammation which plays a crucial role in the development of atherosclerosis (Huang et al., 2023). To evaluate the anti-inflammatory effect of CACN136 in the pathological context of atherosclerosis, the expression levels of IL-6, TNF-α were examined (Figure 7A). Compared to the blank group of mice, the levels of inflammatory factors IL-6 and TNF-α were significantly elevated in the ApoE−/− model group of mice with atherosclerosis. In contrast, the levels of inflammatory factors in the plasma of mice treated with CACN136 and the positive drug Sim decreased significantly compared to the model group, with statistical significance. Furthermore, the levels of inflammatory factors in the plasma of mice treated with CACN136 were lower than those treated with the positive drug Sim, suggesting that CACN136 has a stronger anti-inflammatory effect than Sim. To further confirm the mechanism by which CACN136 inhibits inflammation, immunohistochemical staining for iNOS and Arg1 was performed on the aortic tissues of mice in each group. Compared to the blank group, iNOS was significantly upregulated (Figure 7B), while Arg1 was significantly downregulated (Figure 7C) in the model group, indicating inflammatory infiltration in the aortas of model mice. After CACN136 treatment, the expression of iNOS was significantly downregulated in a concentration-dependent manner, and Arg1 was significantly upregulated in a concentration-dependent manner, consistent with the cellular-level results. These findings suggest that CACN136 can inhibit the inflammatory response in atherosclerosis mice induced by HFD by regulating macrophage polarization.

#### 3.4.2 CACN136 regulated lipid metabolism in HFD-induced ApoE−/− mice

To evaluate the lipid metabolism effect of CACN136 in the pathological context of atherosclerosis, the expression levels of TC, TG, LDL-C, and HDL-C were examined. Due to lipid metabolism disorders, the levels of plasma TC, TG, and LDL-C were significantly higher in the atherosclerosis model group of mice compared to the blank group of mice. After treatment, CACN136 exhibited a significantly better effect in reducing plasma TC, TG, and LDL-C levels compared to the positive drug Sim (Figure 7D). Further, immunohistochemical staining revealed that CACN136 significantly downregulated the expression of CD36 and elevated the expression of ABCG1. (Figures 7E,F), which is consistent with the cellular-level results. These findings indicate that CACN136 corrects lipid metabolism disorders in HFD-induced atherosclerosis mice by simultaneously inhibiting cholesterol intake and increasing cholesterol efflux.

## 4 Discussion

During the last decades, tremendous efforts have been made to improve atherosclerosis. Current treatments for atherosclerosis are mainly chemotherapy and surgical procedures such as recanalization, reconstruction or bypass grafting of stenotic or occluded arteries (Singh et al., 2013; Lordan et al., 2021). Statins, as the current first-line drugs for the treatment of atherosclerosis, have relatively large side effects, and their long-term use can lead to liver enzyme abnormalities, muscle toxicity, and diabetes mellitus, with poor patient compliance (Björnsson, 2017). Therefore, the development of safe and effective new anti-atherosclerosis drugs is particularly necessary and urgent. In recent years, herbal medicines have been deeply accepted and widely used as supplements and alternative therapies for various diseases in many countries (Bergien et al., 2020; Missenda et al., 2023; Zhao et al., 2021). Modern studies have found that curcumin, which is mainly derived from the rhizomes of turmeric-like plants, has pharmacological effects that inhibit atherosclerosis (Si et al., 2015), specifically in terms of reducing inflammation and oxidative stress, anticoagulation, improving lipid metabolism, promoting glucose metabolism, modulating smooth muscle cell proliferation and migration, lowering blood pressure, reducing the number of pathologic neovascularization, and protecting endothelial cells (Peled et al., 2014; Sanson et al., 2013; Moore et al., 2013). Although curcumin has shown some advantages in anti-atherosclerosis, its deficiencies such as poor stability and activity have greatly limited its further developmental applications (Lee et al., 2014; Chen et al., 2019). Zheng found that curcumin analog L3 reduced dyslipidemia and hyperglycemia, attenuated oxidative stress, enhanced the activity of antioxidant enzymes, increased the level of nitric oxide in plasma and aortic arch, and ameliorated adiposity and atherosclerotic degeneration, thereby preventing diabetic atherosclerosis (Kumar et al., 2020). Thus, curcumin analogs may be an effective way to address their deficiencies.

Our group screened and synthesized a series of curcumin analogs in the previous study, in which a novel curcumin analog, CACN136, was found to significantly reduce lipid levels with good safety profile compared with lovastatin positive and negative control groups in hyperlipidemic rats by gavage for 90 days. CACN136 has demonstrated significant antidepressant effects *in vitro* and *in vivo* (Zhao et al., 2021-09), with robust protection against oxidative stress-induced cellular damage. Compared to ascorbic acid and curcumin, CACN136 exhibits superior ABTS radical scavenging capacity (Zhao et al., 2021-09; Chen et al., 2024). Excessive free radicals drive oxidative stress imbalance, a recognized contributor to early atherosclerosis pathogenesis. Notably, oxygen radicals also act as key mediators of inflammation. Emerging evidence supports a causal link between depression and atherosclerosis-related complications (Babic et al., 2023; Bezna et al., 2022; Neil et al., 2017). Collectively, our findings highlight CACN136s therapeutic potential, leveraging its lipid-lowering, antioxidant and anti-inflammatory properties to inhibit AS initiation and progression.

In the early stages of atherosclerosis development, endothelial cells attract monocytes to the arterial wall through chemokine-receptor interactions (Cybulsky and Gimbrone, 1991). Once monocytes migrate to the arterial vascular wall, they differentiate into macrophages, which further polarize due to chronic inflammation. There are two polarization phenotypes: the M1 type, which secretes pro-inflammatory factors such as IL-6 and TNF-α, and the M2 type, which secretes anti-inflammatory factors such as TGF-β. Concurrently, the activity of cholesterol transport pathways increases, leading M1-type macrophages—which mediate pro-inflammatory lesions—to take up large amounts of cholesterol. Subsequently, macrophages increase the expression of various receptors, including scavenger receptors (Al-Hawary et al., 2023). The cholesterol accumulated by scavenger receptors causes macrophages to gradually transform into foam cells, ultimately leading to plaque formation. Thus, macrophages play a crucial role in the development of atherosclerosis. Therefore, this study focuses on macrophages, using a patented compound developed by the research team (Zhao et al., 2021-09), and systematically investigates the anti-atherosclerotic effects and mechanisms of action of the curcumin analog CACN136 through HFD-induced atherosclerosis mouse model, network pharmacology analysis, transcriptomics analysis, and experiments using RAW264.7 macrophages.

Since CACN136 was used for the first time in ApoE−/− mice, we first verified its safety by measuring the blood biochemical indicators and organ HE staining. Safety evaluation results showed that CACN136 did not have a significant detrimental effect on liver function in both the control and ApoE−/− mice, and even exhibited a slight protective effect. On the other hand, Sim showed strong liver toxicity in ApoE−/− mice, indicating a lower safety profile. In the model group, liver tissue structure was disrupted, while the low and medium dose groups showed mild vacuolar degeneration of liver cells. The high dose group did not exhibit significant pathological changes in liver cells. This suggests that the slight liver damage in the low and medium dose groups was not caused by CACN136 itself but rather by liver injury due to the atherosclerosis model. CACN136 has a reparative function, but the reparative effect at low and medium doses is not sufficient. However, when the dosage of CACN136 reached 40 mg/kg, the damaged liver was corrected to a normal level.

We next confirmed the anti-atherosclerosis effect of CACN136. Through Oil Red O staining of aortic root cross-section sections and intact aortas of mice, we found that CACN136 significantly reduced atherosclerotic plaques in mice, and the effect was superior to that of the same dose of simvastatin.

After confirming the anti-atherosclerosis effect of CACN136, we began to predict its mechanism using bioinformatics. The network pharmacology analysis indicated that CACN136 primarily exerted its effects on LPS-induced inflammation and lipid metabolism in the pathological process of atherosclerosis. RAW264.7 macrophage cells are commonly used in atherosclerosis-related inflammation and lipid metabolism studies. Therefore, transcriptomic analysis was conducted to further validate the predictions made by network pharmacology. Among the differentially expressed genes obtained through intersecting analysis, many were related to inflammation and immunity, while fewer were related to lipid metabolism. This could be attributed to the fact that both the control and treatment groups of cells were stimulated with LPS, which primarily mediated the production of inflammation in RAW264.7 cells. However, the KEGG pathway enrichment analysis of the transcriptomic data revealed that lipid and atherosclerosis signaling pathways, in addition to the inflammation signaling pathway still ranked high. Meanwhile, curcumin also has a regulatory effect on inflammation and lipid metabolism (Ji et al., 2021; Hasanzadeh et al., 2020), suggesting that the anti-atherosclerosis mechanism of CACN136 is closely associated with macrophage inflammation and lipid metabolism.

*In vitro*, CACN136 significantly modulates LPS-induced RAW264.7 M1/M2 polarization fractionation and inhibits inflammatory responses. M1 macrophages are a subtype of macrophages in an immunologically activated state. They secrete inflammatory factors such as TNF-α and IL-1β, and exhibit increased expression of iNOS, which is closely associated with promoting inflammatory responses (Wu et al., 2019). iNOS is an important enzyme in M1 macrophages. When macrophages are stimulated by LPS, they produce and release the iNOS protein. In M1 macrophages, the expression of iNOS and the production of NO are significantly increased, thereby promoting inflammatory responses and antimicrobial abilities. M2 macrophages, on the other hand, are a subtype of macrophages in an immunosuppressive state. They secrete immunosuppressive and reparative factors and are closely involved in immune regulation and tissue repair processes. Arg1 is a specific molecular marker in M2 macrophages. The expression and activity of Arg1 increase in M2 macrophages, which can inhibit inflammatory responses, facilitate tissue repair, and regulate immune responses (Luo et al., 2020). Arg1 competitively inhibits the availability of arginine required for the iNOS enzyme, thereby reducing NO synthesis and decreasing inflammatory responses. Some studies have shown that, curcumin can significantly reduce the expression of inflammatory factors in the supernatant of LPS-stimulated raw264.7 cells and inhibits iNOS protein expression with a Macrophage-inducible C-type lectin dependent pattern (Tan et al., 2019). Whereas CACN136, a curcumin analog, both decreased the mRNA and protein levels of IL-1β and iNOS, and increased the mRNA and protein levels of TGF-β and Arg1. This indicates that CACN136 can significantly reduce the number of M1-type RAW264.7 cells and increase the number of M2-type RAW264.7 cells, thereby suppressing inflammatory responses.

*In vitro*, CACN136 inhibits macrophage formation of foam cells while inhibiting RAW264.7 uptake of cholesterol and promoting RAW264.7 efflux of cholesterol. CD36 is capable of binding to LDL, ox-LDL, and other lipid substances, promoting their uptake and conversion into foam cells. Similarly, SRA1 can also bind to ox-LDL and mediate its uptake and internalization. ABCG1 protein is predominantly expressed in macrophages, dendritic cells, and endothelial cells, and it promotes the efflux of cholesterol and lipids (Cheng et al., 2016). ABCA1 protein is mainly expressed in macrophages and endothelial cells, and it plays a role in regulating the transport and metabolism of cholesterol and phospholipids (Chen et al., 2021). ABCA1 facilitates the efflux of cholesterol and phospholipids from the intracellular space to the extracellular space, forming HDL. Additionally, SR-B1 has a high affinity for binding to HDL particles and is involved in the reverse transport and efflux of cholesterol. HDL is considered a “beneficial” cholesterol carrier that promotes reverse transport and efflux of cholesterol. Therefore, CACN136 can reduce the expression of CD36 and SRA1, inhibiting cholesterol internalization, while upregulating the expression of ABCA1, ABCG1, and SR-B1 to promote cholesterol efflux. These findings are similar to curcumin-related reports, such as curcumin enhances the expression of ABCA1 and ABCG1 in foam cells, promotes cholesterol efflux (Xu et al., 2025), regulates oxLDL-induced CD36 expression by inhibiting p38 MAPK phosphorylation in RAW 264.7 cells, and inhibits foam cell formation (Min et al., 2013). This confirms that CACN136 contributes to the reduction of lipid deposition in RAW264.7 macrophages and inhibits the occurrence and development of atherosclerosis.

CACN136 reduced plasma inflammatory factor levels and plasma cholesterol levels in atherosclerosis model mice. The growth curve of mice showed a decrease in body weight in all treatment groups. This is because the drug has a lipid-lowering effect, reducing the levels of cholesterol and triglycerides in mice and subsequently reducing body weight. The rate and extent of weight loss were lower in the positive control group compared to the CACN136 group, indicating that CACN136 degrades mouse fat more rapidly and efficiently, an effect similar to that of curcumin. The hypolipidemic effects of curcumin in rodents were first reported nearly 50 years ago (Rao et al., 1970). Supplementation of curcumin (0.05% w/w) in hamsters fed a high-fat diet significantly reduced plasma TG and free fatty acid concentrations by approximately 25% and 18%, respectively, and increased HDL-C by 17%, but their LDL-C levels did not change significantly (Jang et al., 2008). Another study showed that (0.02% w/w) curcumin-treated mice had significantly lower plasma TC, LDL-C, and TG levels after 18 weeks (Shin et al., 2011). In the present study of atherosclerotic mice treated with CACN136 (0.001% w/w) for only 28 days, the levels of TC, TG and LDL-C were significantly reduced in the treated group compared to the model group. It indicated that the lipid-lowering level of CACN136 was significantly better than curcumin. Measurement of mouse cholesterol levels showed that the plasma HDL levels of AS mice in the model group were higher than those in the treatment groups. This may be due to two reasons (Roth et al., 2020): the therapeutic drug may inhibit the synthesis of high-density lipoprotein in the liver or increase the metabolic rate of HDL, thereby reducing HDL levels in mice (Kühnast et al., 2015; Leão et al., 2019; Tuteja and Rader, 2014); (The, 2023) CACN136 can slow down the progression of atherosclerosis by inhibiting inflammatory responses. However, inhibiting inflammatory responses may also have a negative impact on the synthesis and function of HDL (Van Lenten et al., 1995; Charles-Schoeman et al., 2007), leading to a decrease in HDL levels.

As with the validation results at the cellular level, animal experiments corroborated the specific mechanisms of CACN136 anti-atherosclerosis as: improvement of inflammatory response through downregulation of iNOS and upregulation of Arg1; and improvement of lipid metabolism through upregulation of ABCG1 and downregulation of CD36.

## 5 Conclusion

Our team has identified a novel curcumin analog, CACN136, with superior anti-atherosclerotic activity and safety profile compared to simvastatin. The mechanism involves the regulation of macrophage polarization and lipid metabolism. CACN136 is a promising new drug for future anti-atherosclerosis. However, more in-depth mechanisms remain to be investigated.

## Data Availability

The original contributions presented in the study are included in the article/supplementary material, further inquiries can be directed to the corresponding authors.

## References

[B1] Al-Hawary S. I. S. Jasim S. A. Romero-Parra R. M. Bustani G. S. Hjazi A. Alghamdi M. I. (2023). NLRP3 inflammasome pathway in atherosclerosis: focusing on the therapeutic potential of non-coding RNAs. Pathol. Res. Pract. 246, 154490. 10.1016/j.prp.2023.154490 37141699

[B2] Babic R. Dimitrijevic S. Burazor I. (2023). Association of extent and severity of coronary atheromatosis with clinically undetected depression. Eur. Heart J. 44 (Suppl. ment_2). 10.1093/eurheartj/ehad655.2382

[B3] Bergien S. O. Petersen C. M. Lynning M. Kristiansen M. Skovgaard L. (2020). Use of natural medicine and dietary supplements concomitant with conventional medicine among people with multiple sclerosis. Mult. Scler. Relat. Disord. 44:102197, 10.1016/j.msard.2020.102197 32531752

[B4] Bezna M. C. Pisoschi C. Bezna M. Danoiu S. Tudorascu I. R. Ungureanu L. M. (2022). Immunogenic lipid oxidation by oxidative stress in young people and the risk of early atherogenic endotelial dysfunction. Eur. Heart J. 43 (Suppl. ment_2). 10.1093/eurheartj/ehac544.3077

[B5] Björnsson E. S. (2017). Hepatotoxicity of statins and other lipid-lowering agents. Liver Int. 37 (2), 173–178. 10.1111/liv.13308 27860156

[B6] Charles-Schoeman C. Khanna D. Furst D. E. McMahon M. Reddy S. T. Fogelman A. M. (2007). Effects of high-dose atorvastatin on antiinflammatory properties of high density lipoprotein in patients with rheumatoid arthritis: a pilot study. J. Rheumatol. 34 (7), 1459–1464. Available online at: https://www.jrheum.org/content/34/7/1459.long . 17552046

[B7] Chen J. Wei Y. Li N. Pi C. Zhao W. Zhong Y. (2024). Preliminary investigation into the antidepressant effects of a novel curcumin analogue (CACN136) *in vitro* and *in vivo* . Mol. Neurobiol. 62, 2124–2147. 10.1007/s12035-024-04363-6 39080204

[B8] Chen W. Wang S. Xing D. (2021). New Horizons for the roles and association of APE1/Ref-1 and ABCA1 in atherosclerosis. J. Inflamm. Res. 14, 5251–5271. 10.2147/JIR.S330147 34703267 PMC8526300

[B9] Chen Y. Yang M. Huang W. Chen W. Zhao Y. Schulte M. L. (2019). Mitochondrial metabolic reprogramming by CD36 signaling drives macrophage inflammatory responses. Circ. Res. 125(12):1087–1102. 10.1161/CIRCRESAHA.119.315833 31625810 PMC6921463

[B10] Cheng H. Y. Gaddis D. E. Wu R. McSkimming C. Haynes L. D. Taylor A. M. (2016). Loss of ABCG1 influences regulatory T cell differentiation and atherosclerosis. J. Clin. Invest 126 (9), 3236–3246. 10.1172/JCI83136 27482882 PMC5004951

[B11] Chomczynski P. Sacchi N. (1987). Single-step method of RNA isolation by acid guanidinium thiocyanate-phenol-chloroform extraction. Anal. Biochem. 162 (1), 156–159. 10.1006/abio.1987.9999 2440339

[B12] Cybulsky M. I. Gimbrone M. A. Jr (1991). Endothelial expression of a mononuclear leukocyte adhesion molecule during atherogenesis. Science 251 (4995), 788–791. 10.1126/science.1990440 1990440

[B13] Ference B. A. Ginsberg H. N. Graham I. Ray K. K. Packard C. J. Bruckert E. (2017). Low-density lipoproteins cause atherosclerotic cardiovascular disease. 1. Evidence from genetic, epidemiologic, and clinical studies. A consensus statement from the european atherosclerosis society consensus panel. Eur. Heart J. 38 (32), 2459–2472. 10.1093/eurheartj/ehx144 28444290 PMC5837225

[B14] Hasanzadeh S. Read M. I. Bland A. R. Majeed M. Jamialahmadi T. Sahebkar A. (2020). Curcumin: an inflammasome silencer. Pharmacol. Res. 159, 104921. 10.1016/j.phrs.2020.104921 32464325

[B15] Hou P. Fang J. Liu Z. Shi Y. Agostini M. Bernassola F. (2023). Macrophage polarization and metabolism in atherosclerosis. Cell Death Dis. 14 (10), 691. 10.1038/s41419-023-06206-z 37863894 PMC10589261

[B16] Huang Z. Q. Luo W. Li W. X. Chen P. Wang Z. Chen R. J. (2023). Costunolide alleviates atherosclerosis in high-fat diet-fed ApoE(-/-) mice through covalently binding to IKKβ and inhibiting NF-κB-mediated inflammation. Acta Pharmacol. Sin. 44 (1), 58–70. 10.1038/s41401-022-00928-0 35710877 PMC9813247

[B17] Jang E.-M. Choi M.-S. Jung U. J. Kim M.-J. Kim H.-J. Jeon S.-M. (2008). Beneficial effects of curcumin on hyperlipidemia and insulin resistance in high-fat–fed hamsters. Metabolism 57 (11), 1576–1583. 10.1016/j.metabol.2008.06.014 18940397

[B18] Ji R. Xiang X. Li X. Mai K. Ai Q. (2021). Effects of dietary curcumin on growth, antioxidant capacity, fatty acid composition and expression of lipid metabolism-related genes of large yellow croaker fed a high-fat diet. Br. J. Nutr. 126 (3), 345–354. 10.1017/S0007114520004171 33076999

[B19] Jing J. Guo J. Dai R. Zhu C. Zhang Z. (2023). Targeting gut microbiota and immune crosstalk: potential mechanisms of natural products in the treatment of atherosclerosis. Front. Pharmacol. 14, 1252907. 10.3389/fphar.2023.1252907 37719851 PMC10504665

[B20] Kühnast S. Fiocco M. van der Hoorn J. W. Princen H. M. Jukema J. W. (2015). Innovative pharmaceutical interventions in cardiovascular disease: focusing on the contribution of non-HDL-C/LDL-C-lowering *versus* HDL-C-raising: a systematic review and meta-analysis of relevant preclinical studies and clinical trials. Eur. J. Pharmacol. 763 (Pt A), 48–63. 10.1016/j.ejphar.2015.03.089 25989133

[B21] Kumar A. Gupta P. Rana M. Chandra T. Dikshit M. Barthwal M. K. (2020). Role of pyruvate kinase M2 in oxidized LDL-Induced macrophage foam cell formation and inflammation. J. Lipid Res. 61(3):351–364. 10.1194/jlr.RA119000382 31988148 PMC7053835

[B22] Leão L. Aquino L. A. Dias J. F. Koifman R. J. (2019). Addition of oat bran reduces HDL-C and does not potentialize effect of a low-calorie diet on remission of metabolic syndrome: a pragmatic, randomized, controlled, open-label nutritional trial. Nutrition 65, 126–130. 10.1016/j.nut.2019.03.007 31082790

[B23] Lee S. J. Thien Quach C. H. Jung K. H. Paik J. Y. Lee J. H. Park J. W. (2014). Oxidized low-density lipoprotein stimulates macrophage 18F-FDG uptake *via* hypoxia-inducible factor-1α activation through Nox2-dependent reactive oxygen species generation. J. Nucl. Med. 55(10):1699–1705. 10.2967/jnumed.114.139428 25214643

[B24] Lin K. Chen H. Chen X. Qian J. Huang S. Huang W. (2020). Efficacy of curcumin on aortic atherosclerosis: a systematic review and meta-analysis in mouse studies and insights into possible mechanisms. Oxid. Med. Cell Longev. 2020, 1520747. 10.1155/2020/1520747 31998433 PMC6973199

[B25] Lordan R. Tsoupras A. Zabetakis I. (2021). Platelet activation and prothrombotic mediators at the nexus of inflammation and atherosclerosis: potential role of antiplatelet agents. Blood Rev. 45:100694, 10.1016/j.blre.2020.100694 32340775

[B26] Luo J. He Y. Meng F. Yan N. Zhang Y. Song W. (2020). The role of autophagy in M2 polarization of macrophages induced by micro/nano topography. Int. J. Nanomedicine 15, 7763–7774. 10.2147/IJN.S270100 33116499 PMC7553265

[B27] Lv B. Song G. Jing F. Li M. Zhou H. Li W. (2024). Mortality from cerebrovascular diseases in China: exploration of recent and future trends. Chin. Med. J. Engl. 137 (5), 588–595. 10.1097/CM9.0000000000002760 37415525 PMC10932538

[B28] Maningat P. Gordon B. R. Breslow J. L. (2013). How do we improve patient compliance and adherence to long-term Statin therapy? Curr. Atheroscler. Rep. 15 (1), 291. 10.1007/s11883-012-0291-7 23225173 PMC3534845

[B29] Menon V. P. Sudheer A. R. (2007). Antioxidant and anti-inflammatory properties of curcumin. Adv. Exp. Med. Biol. 595, 105–125. 10.1007/978-0-387-46401-5_3 17569207

[B30] Min K.-J. Um H. J. Cho K.-H. Kwon T. K. (2013). Curcumin inhibits oxLDL-induced CD36 expression and foam cell formation through the inhibition of p38 MAPK phosphorylation. Food Chem. Toxicol. 58, 77–85. 10.1016/j.fct.2013.04.008 23603106

[B31] Missenda M. Morris D. Nault D. (2023). Herbal supplement use for evidence-based indications in US adults: an analysis of national survey data[J]. J. Integr. Complement. Med. 29(9):584–591. 10.1089/jicm.2022.0722 37074703

[B32] Momtazi-Borojeni A. A. Abdollahi E. Nikfar B. Chaichian S. Ekhlasi-Hundrieser M. (2019). Curcumin as a potential modulator of M1 and M2 macrophages: new insights in atherosclerosis therapy. Heart Fail Rev. 24 (3), 399–409. 10.1007/s10741-018-09764-z 30673930

[B33] Moore K. J. Sheedy F. J. Fisher E. A. (2013). Macrophages in atherosclerosis: a dynamic balance. Nat. Rev. Immunol. ,13(10):709–721. 10.1038/nri3520 23995626 PMC4357520

[B34] Nedkoff L. Briffa T. Zemedikun D. Herrington S. Wright F. L. (2023). Global trends in atherosclerotic cardiovascular disease. Clin. Ther. 45 (11), 1087–1091. 10.1016/j.clinthera.2023.09.020 37914585

[B35] Neil S. Huh J. Baronas V. Li X. McFarland H. F. Cherukuri M. (2017). Oral administration of the nitroxide radical TEMPOL exhibits immunomodulatory and therapeutic properties in multiple sclerosis models. Brain Behav. Immun. 62, 332–343. 10.1016/j.bbi.2017.02.018 28238951 PMC5496657

[B36] Peled M. Fisher E. A.(2014). Dynamic aspects of macrophage polarization during atherosclerosis progression and regression. Front. Immunol. 5:579. 10.3389/fimmu.2014.00579 25429291 PMC4228913

[B37] Perry A. S. Dooley E. E. Master H. Spartano N. L. Brittain E. L. Pettee Gabriel K. (2023). Physical activity over the lifecourse and cardiovascular disease. Circ. Res. 132 (12), 1725–1740. 10.1161/CIRCRESAHA.123.322121 37289900 PMC10254078

[B38] Pickett J. R. Wu Y. Zacchi L. F. Ta H. T. (2023). Targeting endothelial vascular cell adhesion molecule-1 in atherosclerosis: drug discovery and development of vascular cell adhesion molecule-1-directed novel therapeutics. Cardiovasc Res. 119 (13), 2278–2293. 10.1093/cvr/cvad130 37595265 PMC10597632

[B39] Rafiee Z. Nejatian M. Daeihamed M. Jafari S. M. (2019). Application of different nanocarriers for encapsulation of curcumin. Crit. Rev. Food Sci. Nutr. 59 (21), 3468–3497. 10.1080/10408398.2018.1495174 30001150

[B40] Rao D. S. Sekhara N. C. Satyanarayana M. N. Srinivasan M. (1970). Effect of curcumin on serum and liver cholesterol levels in the rat. J. Nutr. 100 (11), 1307–1315. 10.1093/jn/100.11.1307 5476433

[B41] Roth G. A. Mensah G. A. Johnson C. O. Addolorato G. Ammirati E. Baddour L. M. (2020). Global burden of cardiovascular diseases and risk factors, 1990-2019: update from the GBD 2019 study. J. Am. Coll. Cardiol. 76 (25), 2982–3021. 10.1016/j.jacc.2020.11.010 33309175 PMC7755038

[B42] Sanson M. Distel E. Fisher E. A. (2013). HDL induces the expression of the M2 macrophage markers arginase 1 and Fizz-1 in a STAT6-dependent process. PLoS One. ,8(8):e74676, 10.1371/journal.pone.0074676 23991225 PMC3749183

[B43] Shin S.-K. Ha T.-Y. McGregor R. A. Choi M.-S. (2011). Long-term curcumin administration protects against atherosclerosis *via* hepatic regulation of lipoprotein cholesterol metabolism. Mol. Nutr. Food Res. 55 (12), 1829–1840. 10.1002/mnfr.201100440 22058071

[B44] Si Y. Guo S. Fang Y. Qin S. Li F. Zhang Y. (2015). Celery seed extract blocks peroxide injury in macrophages *via* Notch1/NF-κB pathway. Am. J. Chin. Med. 43(3):443–455. 10.1142/S0192415X15500287 25916469

[B45] Singh M. Bedi U. S. (2013). Is atherosclerosis regression a realistic goal of Statin therapy and what does that mean? Curr. Atheroscler. Rep.,15(1):294, 10.1007/s11883-012-0294-4 23250630

[B46] Tagde P. Tagde P. Islam F. Tagde S. Shah M. Hussain Z. D. (2021). The multifaceted role of curcumin in advanced nanocurcumin form in the treatment and management of chronic disorders. Molecules 26 (23), 7109. 10.3390/molecules26237109 34885693 PMC8659038

[B47] Tan R.-Z. Liu J. Zhang Y.-Y. Wang H.-L. Li J.-C. Liu Y.-H. (2019). Curcumin relieved cisplatin-induced kidney inflammation through inhibiting Mincle-maintained M1 macrophage phenotype. Phytomedicine 52, 284–294. 10.1016/j.phymed.2018.09.210 30599909

[B48] The W. (2023). Report on cardiovascular health and diseases in China 2022: an updated summary. Biomed. Environ. Sci. 36 (8), 669–701. 10.3967/bes2023.106 37711081

[B49] Tuteja S. Rader D. J. (2014). High-density lipoproteins in the prevention of cardiovascular disease: changing the paradigm. Clin. Pharmacol. Ther. 96 (1), 48–56. 10.1038/clpt.2014.79 24713591

[B50] Van Lenten B. J. Hama S. Y. de Beer F. C. Stafforini D. M. McIntyre T. M. Prescott S. M. (1995). Anti-inflammatory HDL becomes pro-inflammatory during the acute phase response. Loss of protective effect of HDL against LDL oxidation in aortic wall cell cocultures. J. Clin. Invest 96 (6), 2758–2767. 10.1172/JCI118345 8675645 PMC185985

[B51] Wei Y. Zeng M. Pi C. Shen H. Yuan J. Zuo Y. (2022). Novel curcumin derivative-decorated ultralong-circulating paclitaxel nanoparticles: a novel delivery system with superior anticancer efficacy and safety. Int. J. Nanomedicine 17, 5265–5286. 10.2147/IJN.S369761 36406640 PMC9673813

[B52] Wen J. Zhao L. Li Z. Pi C. Feng X. Shi P. (2024). Preparation and anti-colon cancer effect of a novel curcumin analogue (CA8): *in vivo* and *in vitro* evaluation. Front. Pharmacol. 15, 1464626. 10.3389/fphar.2024.1464626 39600365 PMC11589483

[B53] Wu J. J. Yuan X. M. Huang C. An G. Y. Liao Z. L. Liu G. A. (2019). Farnesyl thiosalicylic acid prevents iNOS induction triggered by lipopolysaccharide *via* suppression of iNOS mRNA transcription in murine macrophages. Int. Immunopharmacol. 68, 218–225. 10.1016/j.intimp.2018.12.066 30658315

[B54] Xu M. Ran D. Hu J. Mao J. Qiao D. Zhang Z. (2025). Multifunctional Prussian blue nanozymes alleviate atherosclerosis through inhibiting the inflammation feedback loop. J. Mater. Chem. B 13, 1459–1473. 10.1039/d4tb01926a 39692245

[B55] Zhao J. Zhang Q. Zou G. Gao G. Yue Q. (2021). Corrigendum to arenobufagin, isolated from toad venom, inhibited epithelial-to-mesenchymal transition and suppressed migration and invasion of lung cancer cells *via* targeting IKKβ/NFκB signal cascade. J. Ethnopharmacol. 265:113313, 10.1016/j.jep.2020.113313 32919238

[B56] Zhao L. Wei Y. Pi C. Feng X. Zou Y. Shen H. (2021). inventorsA monocarbonyl analog of curcumin and its preparation and application patent CN113354577A.

[B57] Zhou Y. Chen R. Liu D. Wu C. Guo P. Lin W. (2017). Asperlin inhibits LPS-evoked foam cell formation and prevents atherosclerosis in ApoE(-/-) mice. Mar. Drugs 15 (11), 358. 10.3390/md15110358 29135917 PMC5706047

